# HABP2 is a Novel Regulator of Hyaluronan-Mediated Human Lung Cancer Progression

**DOI:** 10.3389/fonc.2015.00164

**Published:** 2015-07-21

**Authors:** Tamara Mirzapoiazova, Nurbek Mambetsariev, Frances E. Lennon, Bolot Mambetsariev, Joshua E. Berlind, Ravi Salgia, Patrick A. Singleton

**Affiliations:** ^1^Section of Pulmonary and Critical Care, Department of Medicine, Pritzker School of Medicine, The University of Chicago, Chicago, IL, USA; ^2^Section of Hematology/Oncology, Department of Medicine, Pritzker School of Medicine, The University of Chicago, Chicago, IL, USA; ^3^Department of Anesthesia and Critical Care, Pritzker School of Medicine, The University of Chicago, Chicago, IL, USA

**Keywords:** HABP2, hyaluronan, urokinase plasminogen activator, non-small cell lung cancer, transendothelial extravasation

## Abstract

**Background:**

Lung cancer is a devastating disease with limited treatment options. Many lung cancers have changes in their microenvironment including upregulation of the extracellular matrix glycosaminoglycan, hyaluronan (HA), which we have previously demonstrated can regulate the activity of the extracellular serine protease, hyaluronan binding protein 2 (HABP2). This study examined the functional role of HABP2 on HA-mediated human lung cancer dynamics.

**Methods:**

Immunohistochemical analysis was performed on lung cancer patient samples using anti-HABP2 antibody. Stable control, shRNA, and HABP2 overexpressing human lung adenocarcinoma cells were evaluated using immunoblot analysis, migration, extravasation, and urokinase plasminogen activator (uPA) activation assays with or without high-molecular weight HA or low-molecular weight HA (LMW-HA). In human lung cancer xenograft models, primary tumor growth rates and lung metastasis were analyzed using consecutive tumor volume measurements and nestin immunoreactivity in nude mouse lungs.

**Results:**

We provide evidence that HABP2 is an important regulator of lung cancer progression. HABP2 expression was increased in several subtypes of patient non-small cell lung cancer samples. Further, HABP2 overexpression increased LMW-HA-induced uPA activation, migration, and extravasation in human lung adenocarcinoma cells. *In vivo*, overexpression of HABP2 in human lung adenocarcinoma cells increased primary tumor growth rates in nude mice by ~2-fold and lung metastasis by ~10-fold compared to vector control cells (*n* = 5/condition).

**Conclusion:**

Our data suggest a possible direct effect of HABP2 on uPA activation and lung cancer progression. Our observations suggest that exploration of HABP2 in non-small cell lung carcinoma merits further study both as a diagnostic and therapeutic option.

## Introduction

Effective therapeutic strategies for lung cancer, the leading cause of cancer-associated mortality worldwide, are extremely limited exemplifying the need for novel therapeutic targets (1, 2). Previous reports have demonstrated that the extracellular serine protease, hyaluronan binding protein 2 (HABP2), is upregulated in several types of human non-small cell lung cancer (NSCLC) (3). However, the functional consequences of HABP2 overexpression in NSCLC are poorly defined. HABP2, also called factor VII activating protease (FSAP), is an extracellular serine protease involved in the extrinsic pathway of blood coagulation via activation of factor VII and fibrinolysis via activation of pro-urokinase type plasminogen activator (pro-uPA) (4, 5). HABP2 has been implicated in several disease processes including atherosclerosis and deep venous thrombosis (4, 6). Initially expressed in a single amino acid chain proenzymatic form, HABP2 undergoes autocatalytic cleavage upon binding of a ligand (7). Originally isolated based on its affinity for hyaluronan (HA) (8), HABP2 is capable of being activated by variety of polyanions including heparin and nucleic acids (9, 10). The fully mature enzyme consists of trypsin-like catalytic domain, linked via disulfide bond to the kringle domain and three epidermal growth factor (EGF)-like domains (4, 5). The second and third EGF-like domains form the polyanion binding domain (PABD) (10). We have previously reported that HABP2 is upregulated in the pulmonary vasculature with acute lung injury (ALI) and promotes disruption of vascular integrity (11).

The major non-sulfated glycosaminoglycan in most tissues, hyaluronan (HA), plays an important role in cancer progression (12–17). HA is composed of a linear repeat of disaccharide units consisting of d-glucuronic acid and *N*-acetylglucosamine (18–21). The major form of HA *in vivo*, high-molecular weight HA (HMW-HA), has a molecular weight >1 million Da (17). HMW-HA exhibits a random coil structure that can expand in aqueous solutions (22). During disease states, HMW-HA can be cleaved to low-molecular weight HA (LMW-HA) via hyaluronidase enzymes and reactive oxygen species (ROS) (23–29). We have previously reported that HMW-HA inhibits, while LWM-HA enhances, HABP2 enzymatic activity (11).

In this study, we investigated the effects of stable silencing or overexpression of HABP2 on HMW-HA and LMW-HA-regulated human NSCLC cell migration, extravasation, tumor growth, and metastasis. Here, we present evidence of a role for HABP2 in the regulation of urokinase plasminogen activator (uPA) activation during lung cancer progression. Further, through the use of tumor xenograft models, our results suggest a possible therapeutic role for HABP2 antagonism on cancer growth and metastasis, which merits further research.

## Materials and Methods

### Cell culture and reagents

Human NSCLC cell lines A549, SK-LU-1, H1703, H358, H1993, H661, SW1573, H522, H226, H1437, H1838, H1975, H2170, and non-cancerous BEAS-2B were obtained from ATCC (Walkersville, MD, USA) and cultured in Roswell Park Memorial Institute complete medium (Cambrex, East Rutherford, NJ, USA) at 37°C in a humidified atmosphere of 5% CO_2_, 95% air, with passages 6–10 used for experimentation. Unless otherwise specified, reagents were obtained from Sigma (St. Louis, MO, USA). UK122 was purchased from Santa Cruz Biotechnology (Santa Cruz, CA, USA). Reagents for SDS-PAGE electrophoresis were purchased from Bio-Rad (Richmond, CA, USA) and Immobilon-P transfer membrane was purchased from Millipore (Millipore Corp., Bedford, MA, USA). Mouse anti-HABP2 antibody was purchased from Novus Biologicals (Littleton, CO, USA). Mouse anti-human nestin antibody that does not react with mouse or rat nestin (clone 10C2) was purchased from Millipore (Bedford, MA, USA). Mouse anti-β-actin antibody was purchased from Sigma (St. Louis, MO, USA). Secondary horseradish peroxidase-labeled antibodies were purchased from Amersham Biosciences (Piscataway, NJ, USA).

### Immunoblotting

Immunoblotting was performed, as we have previously described (24, 30). Cellular materials from treated or untreated human NSCLC cells were incubated with lysis buffer [50 mM HEPES (pH 7.5), 150 mM NaCl, 20 mM MgCl_2_, 1% Triton X-100, 0.1% SDS, 0.4 mM Na_3_VO_4_, 40 mM NaF, 50 μM okadaic acid, 0.2 mM phenylmethylsulfonyl fluoride, and 1:250 dilution of Calbiochem protease inhibitor mixture 3). The samples were then run on SDS-PAGE in 4–15% polyacrylamide gels, transferred onto Immobilon™ membranes, and developed with specific primary and secondary antibodies. Visualization of immunoreactive bands was achieved using enhanced chemiluminescence (Amersham Biosciences, Piscataway, NJ, USA). In some instances, immunoreactive bands were quantitated using computer-assisted densitometry.

### Preparation of HMW-HA and LMW-HA

High-molecular weight HA and LMW-HA were prepared similar to that as we have previously described (24). For HMW-HA, 500 mg of hyaluronan sodium salt from *Streptococcus zooepidemicus* was centrifuged in an Ultrafree-MC™ Millipore 100 kDa MW cutoff filter and the upper (non-flow-through) portion was kept and resuspended in PBS, pH = 7.4. For LMW-HA, 500 mg of hyaluronan sodium salt from *S. zooepidemicus* was digested with 20,000 U of bovine testicular hyaluronidase (Type VI-S), lyophilized powder, 3,000–15,000 U/mg (Sigma, H3631) in digestion buffer (0.1M sodium acetate, pH 5.4, 0.15M NaCl) for 24 h, and the reaction stopped with 10% trichloroacetic acid. The resulting solution was centrifuged in an Ultrafree-MC™ Millipore 5 kDa MW cutoff filter and the flow through was dialyzed against distilled water for 24 h at 4°C in 500 Da cutoff Spectra-Por tubing (Pierce-Warriner, Chester, UK). HMW-HA and LMW-HA were quantitated using an ELISA-like competitive binding assay with a known amount of fixed HA and biotintylated HA-binding peptide (HABP) as the indicator (Echelon Inc.). HA solutions were filtrated through 0.22 μm filters and kept in sterile tubes. In some cases, both LMW-HA and HMW-HA were subject to boiling, proteinase K (50 μg/ml) digestion, hyaluronidase SD digestion [*Streptococcus dysgalactiae*, NorthStar Bioproducts Associates of Cape Cod Inc., East Falmouth, MA, USA (100741-1A), 100 mU/ml utilized], or addition of boiled (inactivated) hyaluronidase SD to test for possible protein/lipid contaminants. To test for endotoxin contamination of HA, a lipopolysaccharides (LPS) BioAssay ELISA kit (USBiological Life Sciences) was utilized. LMW-HA with HA standards (Sigma and Enzo Life Sciences) were run on 4–20% Tris/Borate/EDTA (TBE) gels and stained with Stains-All (Sigma) to confirm LMW-HA purity and size.

### Stable vector control and HABP2 overexpression in NSCLC cells

The pCMV6-XL5 human HABP2 overexpression vector and vector control (pCMV6-AC-GFP) were purchased from Origene. SK-LU-1 cells were transfected with vectors using FuGENE HD™ as the transfection reagent (Roche Applied Sciences) according to the protocol provided by Roche, as we have previously described (31). Cells (~40% confluent) are serum-starved for 1 h followed by incubated with vectors for 6 h in serum-free media. Serum-containing media was then added (10% serum final concentration) for 42 h and G418 selection reagent was added. Overexpression was confirmed by immunoblot analysis with anti-HABP2 antibody (Novus Biologicals, Littleton, CO, USA).

### Stable control and HABP2 small hairpin RNA transfection in human NSCLC cells

Stable Control and HABP2 shRNA (Santa Cruz Biotechnology, Santa Cruz, CA, USA) were stably transfected into SK-LU-1 cells, as we have previously described (32). Cells (~40% confluent) were serum-starved for 1 h followed by incubated with shRNA for 6 h in serum-free media. Serum-containing media was then added (10% serum final concentration) for 42 h and puromycin selection reagent was added. Inhibition of protein expression was confirmed by immunoblot analysis with anti-HABP2 antibody (Novus Biologicals, Littleton, CO, USA).

### uPA activation assay

Activation of uPA was quantitated using an uPA Activity Assay Kit (Chemicon International, Temecula, CA, USA). Briefly, serum-free media from control, HABP2 silenced or HABP2 overexpressing SK-LU-1 cells treated with either 100 nM HMW-HA or LMW-HA for 6 h was added to a 96 well microplate and a chromogenic substrate, which is cleaved by active uPA was added. The resulting colorimetric assay was read at an optical density of 405 nm in a microplate reader. Each assay was set up in triplicate and repeated at least five times.

### Human NSCLC cell migration assay

Measurement of *in vitro* NSCLC cell migration was performed, as we have previously described (30). Twenty-four transwell units with 8 μm pore size (Millipore, Billerica, MA, USA) were used for monitoring *in vitro* cell migration. Control or HABP2 overexpressing cells (5 × 10^3^ cells/well) were plated in the upper chamber and incubated with 0.2 ml of serum-free media containing either vehicle (control), 100 nM LMW-HA or 100 nM HMW-HA with or without 1 h pretreatment with 1 μM of the uPA inhibitor UK122 and media with serum was added to the lower chamber. Cells were allowed to migrate through the pores for 18 h. Cells from the upper and lower chamber were quantitated using the CellTiter96™ MTS assay (Promega, San Luis Obispo, CA, USA) and read at 492 nm. Percent migration was defined as the number of cells in the lower chamber divided by the number of cells in both the upper and lower chamber. Each assay was set up in triplicate and repeated at least five times.

### Cell motion analysis on Imaris

The Olympus LCV110U VivaView was used for long-term live cell imaging. Cells were recorded for 10 s every 10 min for 24 h. After record, images were processed and converted into a TIFF format using ImageJ. Stacks of high-resolution images were converted to AVI video as the full trace of the records. To analyze cell track and velocity, imaging data were processed with Imaris (Bitplane). Imaris spot detection module and Brownian algorithm were used to calculate cell coordinates (mean position) over time. Due to cell clustering, some compromises were made between the number of cells tracked and the quality of the traces. The results of the tracking were manually edited to correct or remove errant traces. The data generated by Imaris were then exported to an Excel worksheet and analyzed to calculate cell track and speed. Ten cells per condition were utilized.

### Transendothelial extravasation assay

The ability of NSCLC cells to invade though a layer of endothelial cells (ECs) was quantified using transendothelial monolayer resistance measurements using an electrical substrate-impedence sensing system (Applied Biophysics, Troy, NY, USA), as we have previously described (31). Briefly, human pulmonary microvascular ECs were grown to confluence on gold plated microelectrodes connected to a phase-sensitive lock-in amplifier. Stable vector control, HABP2 silenced, or HABP2 overexpressing SK-LU-1 cells (5 × 10^3^ cells/well) untreated or treated with 1 μM UK122 or 5% serum media only control were added to the confluent endothelial monolayers on the electrodes. The electrical substrate-impedence sensing system allows for continuous measurement of the endothelial monolayer resistance as the SK-LU-1 cells attach and begin to invade into the monolayer. A decrease in transendothelial monolayer resistance indicates a disrupted endothelial monolayer barrier via transendothelial extravasation of NSCLC cells. Resistance readings were normalized relative to an undisturbed confluent endothelial monolayer. Experiments were performed in triplicate with five independent experiments.

### Human NSCLC Xenograft studies in nude mice

All animal procedures were carried out in accordance with the guidelines provided by the Institutional Animal Care and Use Committee of the University of Chicago (Chicago, IL, USA). All mice were 8- to 12-week-old males obtained from Harlan Laboratories (Indianapolis, IN, USA). At the end of each experiment, lungs and primary tumors were collected, fixed in formalin, and embedded in paraffin or solubilized in extraction buffer for immunoblot analysis. 1.0 × 10^6^ stable vector control, HABP2 silenced, or HABP2 overexpressing SK-LU-1 cells were mixed with Matrigel supplemented with 100 ng/ml HMW-HA or LMW-HA (conditions we previously established to produce optimal SK-LU-1 tumor growth) and injected subcutaneously into the flank of Ncr-nude mice. Tumor nodules were measured regularly for 30 days using calipers, and tumor volume V_T_ (cubic millimeter) was calculated using the ellipsoid formula *A*^2^ × *B* × π/6, where *A* represents the smaller diameter (33). Five mice per condition were utilized.

### Quantification of lung metastasis

To characterize metastasis from primary tumors growing in mouse hind flank, lungs from vector control, HABP2 silenced, or HABP2 overexpressing SK-LU-1 hind flank-injected mice were solubilized in extraction buffer [50 mM HEPES (pH 7.5), 150 mM NaCl, 20 mM MgCl_2_, 1% Triton X-100, 0.2% SDS, 0.4 mM Na_3_VO_4_, 40 mM NaF, 50 μM okadaic acid, 0.2 mM phenylmethylsulfonyl fluoride, 1:250 dilution of Calbiochem protease inhibitor mixture 3] with sonication. The resulting material was run on SDS-PAGE in 4–15% polyacrylamide gels, transferred onto Immobilon™ membranes, and developed with specific primary and secondary antibodies. An anti-human nestin antibody (clone 10C2, Millipore) that does not react with mouse or rat nestin was used for quantitation of SK-LU-1 cell metastasis to the lung (32). Five mice per condition were utilized.

### Human lung immunohistochemistry

Immunohistochemical analysis was performed on human tissue microarrays (TMA, RayBiotech, Inc., Norcross, GA, USA). For antigen retrieval, sections were heated in Tris-EDTA buffer (pH = 9) for 10 min and incubated for 1 h at room temperature with mouse anti-HABP2 antibody (1:100) (Novus Biologicals, Littleton, CO, USA). This was followed by 30 min incubation with goat anti-mouse HRP-conjugated IgG (EnVisionTM+, Dako). Slides were developed for 5 min with 3,3′-diaminobenzidene chromogen and counterstained with hematoxylin. Five samples per condition were utilized.

### Quantification of HABP2 expression in human lung samples

Hyaluronan binding protein 2 quantification in human lung cancer samples was performed, as we have previously described (32). HABP2 immunohistochemical staining was performed on tissue microarrays (TMA, RayBiotech, Inc., Norcross, GA, USA) containing 10 cases of human bronchioloalveolar carcinoma (BAC), adenocarcinoma (Adeno) or squamous cell carcinoma (SCC) lung cancer, and adjacent normal tissue. The microscopic images of each slide were scanned and analyzed using Chromavision Automated Cellular Imaging System (ACIS, Clarient, Aliso Viejo, CA, USA) in a digital format that accounts for all individual microscopic fields. Areas of interest were outlined and the intensity was quantified by the software and converted to a score over a set of defined parameters. Brown integrated optical density (IOD)/10 μ^2^ was used as a unit of measurement since IOD (intensity multiplied by brown area in squared micrometers). Five samples per condition were utilized.

### Statistical analysis

Results are expressed as mean ± SD of five independent experiments. For data analysis, experimental samples were compared to controls by unpaired Student’s *t*-test. For multiple-group comparisons, a one-way variance analysis (ANOVA) and *post hoc* multiple comparisons tests were used. Differences between groups were considered statistically significant when *p* value was <0.05. All statistical analyses were performed using the GraphPad Prism program (GraphPad Software Inc., USA).

## Results

**Figure 1:**
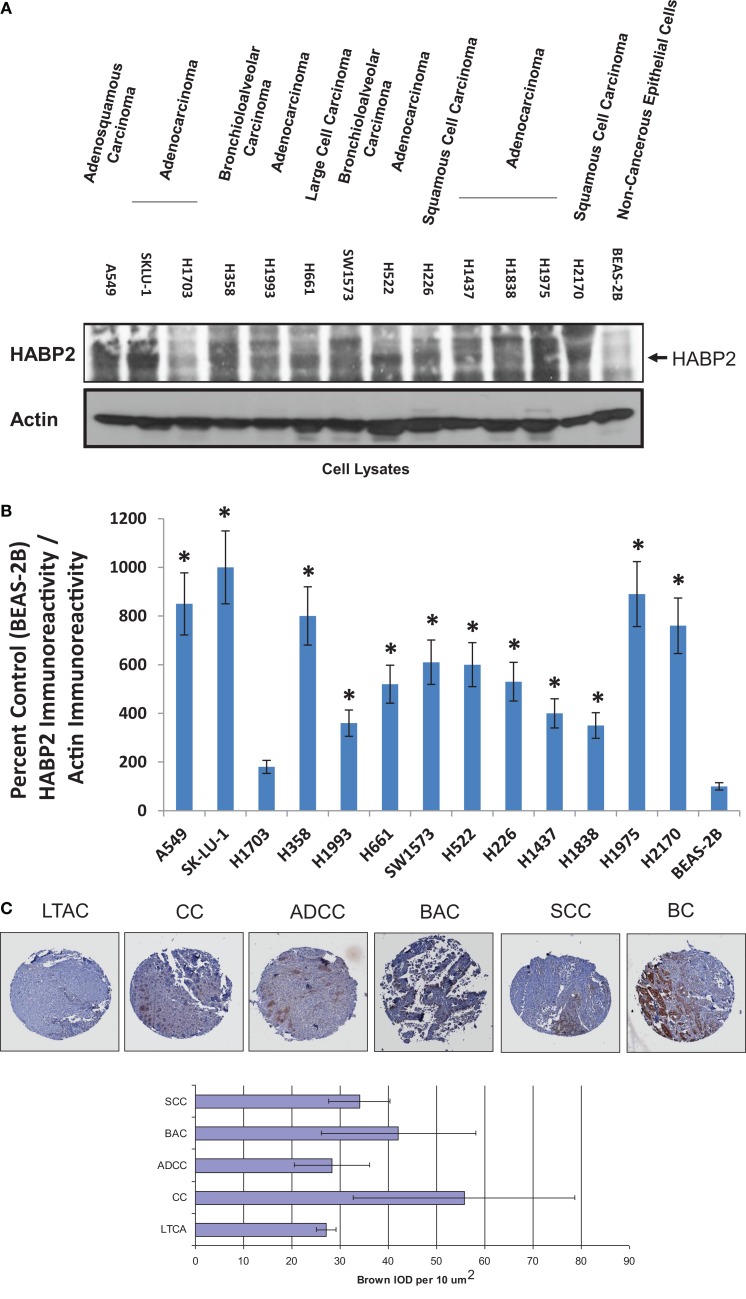
**Hyaluronan binding protein 2 is upregulated in several human NSCLC cell lines and patient tumors**. **(A)** Immunoblot analysis using anti-HABP2 and anti-actin antibodies of cell lysates from human adenosquamous carcinoma (A549), adenocarcinoma (SKLU-1, H1703, H1993, H522, H1437, H1838, H1975), bronchioloalveolar carcinoma (H358, SW1573), large cell carcinoma (H661), squamous cell carcinoma (H226, H2170), and non-cancerous BEAS-2B cells. The arrow indicates the position of HABP2. **(B)** Due to differences in the loading controls, we performed computer-assisted densitometry on the immunoreactive bands in **(A)**. The results are graphically displayed as percent control (BEAS-2B) HABP2 immunoreactivity divided by actin immunoreactivity with error bars = SD. The single asterisks (*) refers to a statistically significant difference (*p* < 0.05) from control (BEAS-2B). **(C)** Representative immunohistochemical (IHC) analysis of normal lung and lung tumor samples from patients indicating a trend toward increased expression of HABP2 in lung carcinoid cancer (CC), bronchioloalveolar carcinoma (BAC), adenocarcinoma (ADCC), squamous cell carcinoma (SCC), and breast cancer (BC) compared to lung tissue adjacent to cancer (LTAC). HABP2-specific brown staining intensity from five patient samples per group were analyzed, as we have previously described.

Non-small cell lung cancer, which accounts for ~80% of all lung cancers, is heterogeneous disease composed of several types, including adenocarcinoma, BAC, SCC, adenosquamous carcinoma, and large cell carcinoma (34, 35). We determined the relative levels of HABP2 expression in human NSCLC cell lines representing these various types and observed a 5- to 10-fold increase in HABP2 expression is most cell lines relative to control non-cancerous BEAS-2B cells (see Figure 1A). These results are consistent with previous published data indicating that the HABP2 is upregulated in lung tissue from patients with NSCLC (3). Due to differences in the loading controls, we performed computer-assisted densitometry on the immunoreactive bands that are graphically displayed in Figure 1B. We next examined HABP2 expression in lung samples from patients with several forms of NSCLC. Figure 1C shows the immunohistochemical scoring of HABP2 in LTAC, lung tissue adjacent to cancer; CC, lung carcinoid cancer; ADCC, lung adenocarcinoma; BAC, bronchoalveolar carcinoma; SCC, lung small cell cancer; BC, breast cancer (TMA slide from RayBiotech, Inc.) (32). While there was considerable variability as indicated by the error bars possibly due to our low sample size (*N* = 5/condition), we observed a trend toward increased HABP2 expression in several types of NSCLC compared to lung tissue adjacent to cancer.

**Figure 2:**
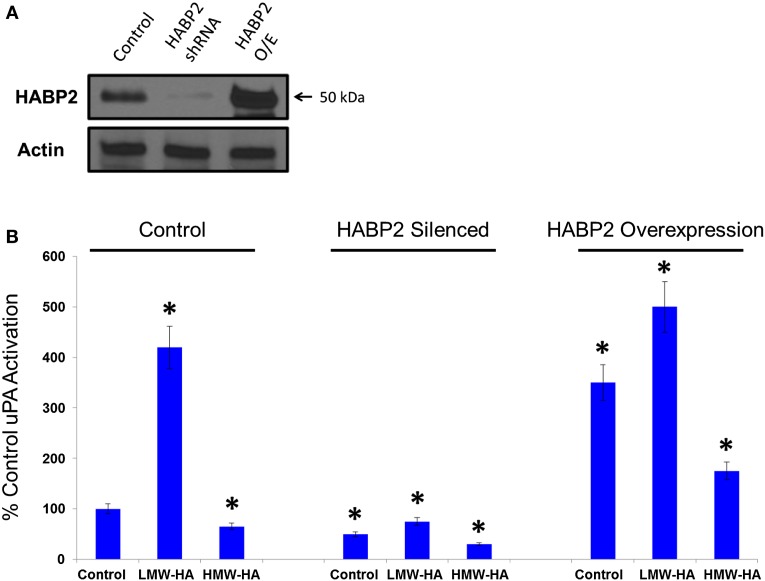
**Low-molecular weight HA and HABP2 promote uPA activation in human lung adenocarcinoma cells**. **(A)** Immunoblot analysis of control, HABP2 shRNA, and HABP2 overexpressing (O/E) SK-LU-1 cells using anti-HABP2 and anti-actin antibodies. **(B)** Graphical representation of uPA activation from media of treated SK-LU-1 cells. Control, HABP2 shRNA, and HABP2 overexpressing cells were either untreated or treated with 100 nM of either LMW-HA or HMW-HA for 6 h in serum-free media. Media was then collected and analyzed using an uPA Activity Assay Kit (Chemicon International, Temecula, CA, USA). The single asterisks (*) refers to a statistically significant difference (*p* < 0.05) from control (100%). Each assay was set up in triplicate and repeated at least five times.

To test the functional significance of HABP2 upregulation, we generated stable vector control and HABP2 overexpressing human SK-LU-1 lung adenocarcinoma cells. We chose the SK-LU-1 cell line to study HABP2 overexpression based on its low-basal metastatic potential (36). We observe robust HABP2 immunoreactivity in stable overexpressing HABP2 and significant silencing of expression in shRNA treated SK-LU-1 cells compared to control (Figure 2A). Since there is an intimate relationship between HABP2 and uPA often leading to reciprocal protease activation (7), we next focused on measuring uPA activity, which has been reported to contribute to lung cancer progression. We have previously reported that HMW-HA (the main form of HA in the body) inhibits HABP2 activation while LMW-HA (generated in disease states, such as cancer, by hyaluronidase enzymes and ROS) activates HABP2 activity (11), and therefore, we examined the roles of HABP2 and HA on uPA activation in SK-LU-1 cells (Figure 2B). Our results indicate that HABP2 overexpression promotes, while HABP2 silencing inhibits basal uPA activity. In addition, LMW-HA treatment enhances this activity, while HMW-HA addition tends to inhibit uPA activation in SK-LU-1 cells. Considering the low levels of uPA activity in HABP2 silenced cells, we focused our consequent studies on comparing stable vector control and HABP2 overexpressing SK-LU-1 functional analysis.

**Figure 3:**
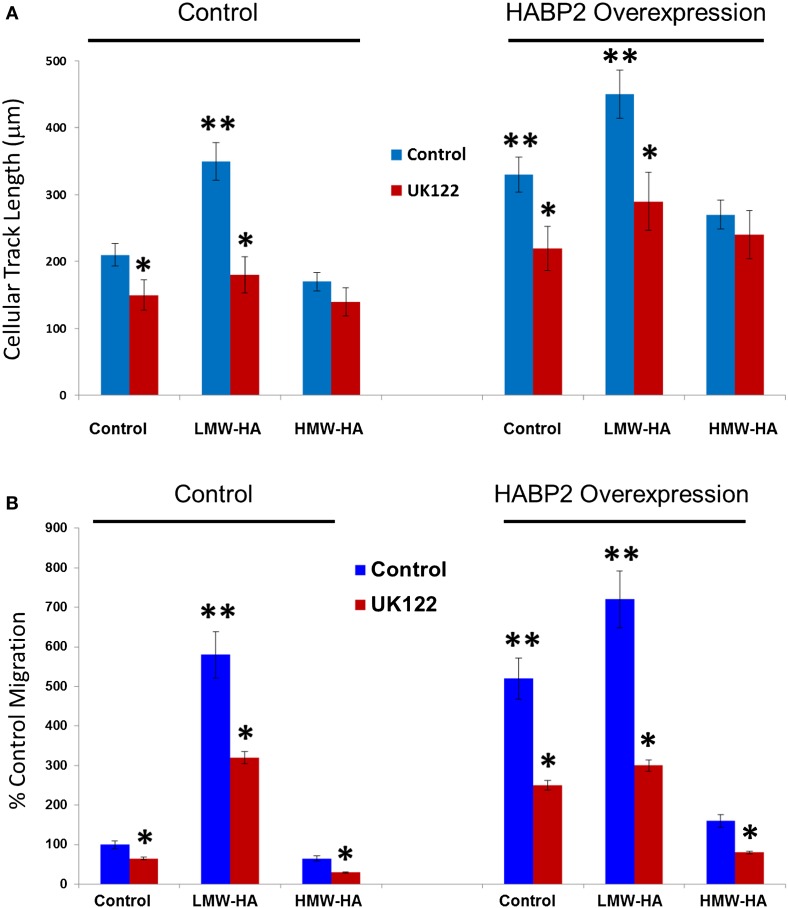
**Low-molecular weight HA and HABP2 promote uPA-dependent SK-LU-1 cell motion and migration**. **(A)** Graphical representation of cell motion as measured by cellular track length (micrometer) from treated SK-LU-1 cells. Stable vector control or HABP2 overexpressing SK-LU-1 cells were seeded (30 × 10^3^ cells/dish) to glass bottom dishes (MatTek), either pretreated with vehicle or 1 μM uPA inhibitor (UK122) for 1 h and either no (control) or 100 nM of LMW-HA or HMW-HA was then added. Cells were then processed for video recording for 24 h. Ten cells for each treatment were manually labeled and cellular track length was then analyzed using Imaris software. The single asterisks (*) represents a statistically significant difference (*p* < 0.05) from control (no UK122 addition). The double asterisks (**) represents a statistically significant difference (*p* < 0.05) from control (200 nm cellular track length). Each assay was set up in triplicate and repeated at least five times. **(B)** Graphical representation of the percent control migration of treated SK-LU-1 cells. Control or HABP2 overexpressing cells (5 × 10^3^ cells/well) were plated on the upper chamber of 8 μm transwell units and incubated with 0.2 ml of serum-free media containing either vehicle (control), 100 nM LMW-HA or 100 nM HMW-HA with or without 1 h pretreatment with 1 μM of the uPA inhibitor UK122 and media with serum was added to the lower chamber. Cells were allowed to migrate through the pores for 18 h. Cells from the upper and lower chamber were quantitated using the CellTiter96™ MTS assay (Promega, San Luis Obispo, CA, USA) and read at 492 nm. Percent migration was defined as the number of cells in the lower chamber divided by the number of cells in both the upper and lower chamber. The single asterisks (*) represents a statistically significant difference (*p* < 0.05) from control (no UK122 addition). The double asterisks (**) represents a statistically significant difference (*p* < 0.05) from control (100%). Each assay was set up in triplicate and repeated at least five times.

We next examined HA- and HABP2-regulated SK-LU-1 human lung adenocarcinoma cell motion and migration (Figure 3). Using Cell Motion Analysis on Imaris (see Materials and Methods), individual cell movement over the time was analyzed (Figure 3A). Ten cells for each treatment were manually labeled. Spots were tracked over time and total maximal distance for all spots was recorded for 24 h. Cellular track length was measured in microns. HABP2 overexpression or treatment with LMW-HA (but not HMW-HA) significantly increased cellular track length (double asterisks) compared to vector control, which were significantly inhibited by the uPA antagonist UK122 (37) (single asterisks). We observed similar results with SK-LU-1 migration using 8 μm transwell units (see Materials and Methods) (Figure 3B) indicating that treatment with LMW-HA and HABP2 overexpression significantly increase SK-LU-1 cell motion and migration in an uPA-dependent manner.

**Figure 4:**
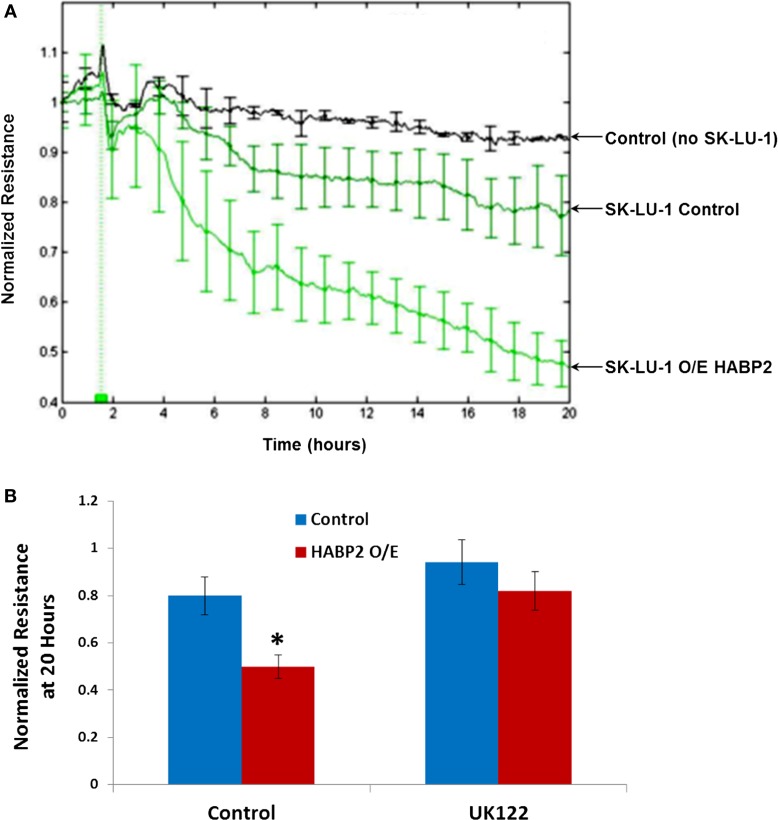
**Hyaluronan binding protein 2 overexpression in human lung adenocarcinoma cells promotes uPA-dependent transendothelial extravasation**. **(A)** Graphical representation of the ability of vector control and HABP2 overexpressing (O/E) SK-LU-1 cells to disrupt a confluent human pulmonary microvascular endothelial cell monolayer using an electrical substrate-impedence sensing system, as we have previously described. This system continuously measures endothelial monolayer resistance as the SK-LU-1 cells attach and begin to invade into the monolayer. A decrease in resistance indicates a disrupted endothelial monolayer barrier via transendothelial extravasation of the NSCLC cells. HABP2 overexpressing SK-LU-1 cells have increased extravasation properties compared to vector control cells, which becomes apparent ~2–4 h after NSCLC cell addition (vertical green line) to the confluent endothelial monolayer with five pooled-independent experiments repeated in triplicate and error bars = SD. **(B)** Graphical representation of normalized resistance at 20 h with vector control and HABP2 overexpressing (O/E) SK-LU-1 cells with or without pretreatment of 1 μM uPA inhibitor (UK122) for 1 h prior to addition to confluent human pulmonary microvascular endothelial monolayers. The single asterisks (*) represents a statistically significant difference (*p* < 0.05) from control.

In order for many tumor cells to metastasize, they need to breech the endothelium to enter the bloodstream (12). We tested the *in vitro* ability of vector control and HABP2 overexpressing SK-LU-1 cells to disrupt a confluent human pulmonary microvascular EC monolayer using an electrical substrate-impedence sensing system, as we have previously described (31). This system continuously measures endothelial monolayer resistance as the SK-LU-1 cells attach and begin to invade into the monolayer. A decrease in resistance indicates a disrupted endothelial monolayer barrier via transendothelial extravasation of the NSCLC cells. Figure 4 indicates HABP2 overexpressing SK-LU-1 cells have increased extravasation properties compared to vector control cells, which becomes apparent ~2–4 h after NSCLC cell addition to the confluent endothelial monolayer. These effects are inhibited by the uPA inhibitor UK122 (Figure 4B), results similar to our *in vitro* cell motion and migration assays.

**Figure 5:**
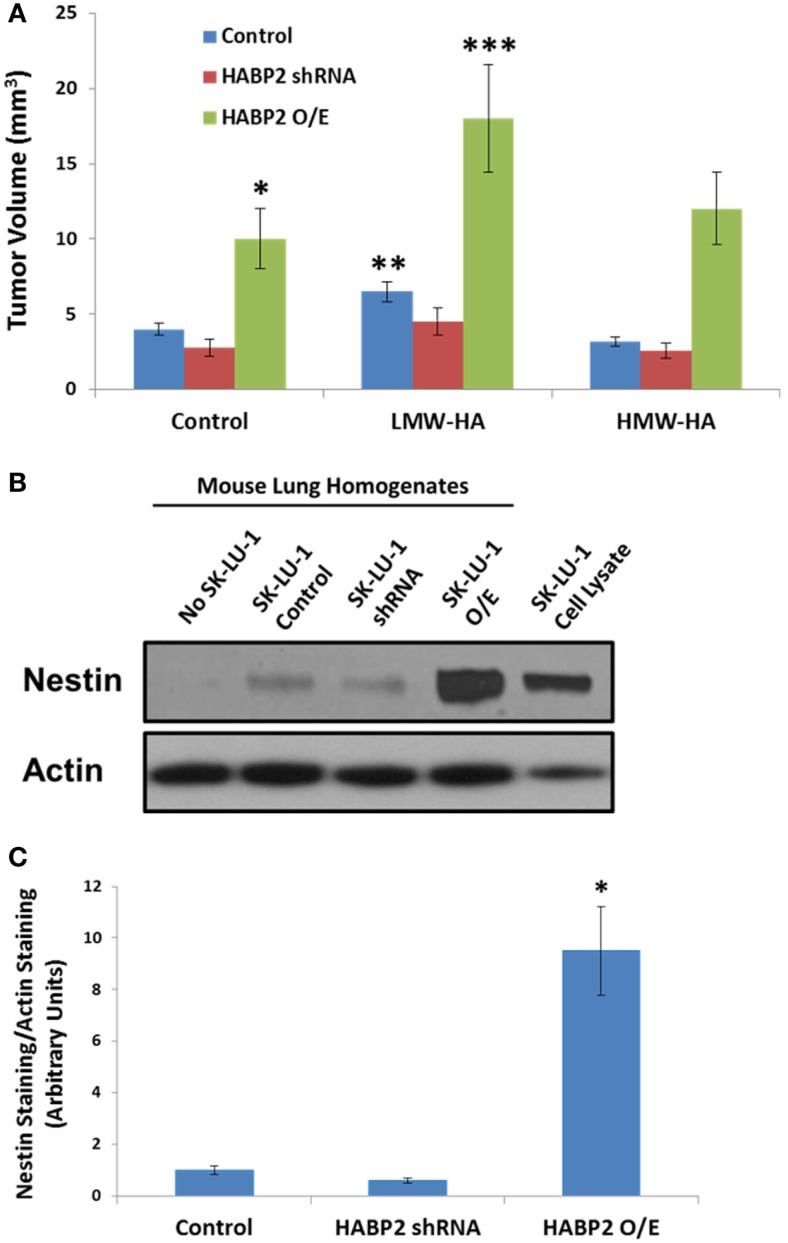
**Hyaluronan binding protein 2 promotes human lung adenocarcinoma tumor growth and metastasis**. **(A)** Graphical representation tumor volume of vector control, HABP2 shRNA, and HABP2 overexpressing (O/E) SK-LU-1 cells embedded in Matrigel supplemented with 100 ng/ml HMW-HA or LMW-HA injected into the hind flank of nude mice for 30 days. The single asterisks (*) represents a statistically significant difference (*p* < 0.05) between control and HABP2 overexpressing (O/E) SK-LU-1 tumors. The double asterisks (**) represents a statistically significant difference (*p* < 0.05) between control and LMW-HA added SK-LU-1 tumors. The triple asterisks (***) represents a statistically significant difference (*p* < 0.05) between control HABP2 overexpressing (O/E) tumors and LMW-HA added HABP2 overexpressing (O/E) SK-LU-1 tumors. **(B)** Representative immunoblot analysis of human SK-LU-1 NSCLC cells alone, control nude mouse lung homogenate, lung homogenate from nude mouse with vector control SK-LU-1 hind flank tumor or lung homogenates from nude mice with HABP2 shRNA or HABP2 overexpressing (O/E) SK-LU-1 hind flank tumors using anti-nestin or anti-actin antibodies. **(C)** Graphical representation of lung metastasis from vector control or HABP2 overexpressing SK-LU-1 hind flank tumors in nude mice using quantitation of nestin immunoreactivity as depicted in **(B)**. The asterisks (*) represents a statistically significant difference (*p* < 0.05) between HABP2 overexpressing (O/E) versus control or HABP2 shRNA with *n* = 5/group.

We next translated our *in vitro* results to an *in vivo* human NSCLC xenograft model. Vector control, HABP2 silenced, and HABP2 overexpressing SK-LU-1 cells were mixed with Matrigel supplemented with 100 ng/ml HMW-HA or LMW-HA and were injected into the hind flank of nude mice. Tumor volumes were measured using calipers for 30 days (see Figure 5A) as described in the Section “Materials and Methods.” HABP2 overexpressing SK-LU-1 cells had ~2-fold increase in primary tumor growth rate compared to vector control cells (single asterisks). HABP2 silenced cells did not show a significant tumor size difference from control. LMW-HA increased control (double asterisks) and HABP2 overexpressing (triple asterisks) in a statistically significant manner. In contrast, HMW-HA had little additional effect on tumor growth.

Considering the differential growth rates of the primary tumors in nude mice, we next examined the metastatic potential of vector control and HABP2 overexpressing SK-LU-1 primary flank tumors. These tumor cells tend to have low basal metastatic potential (36). However, as time progresses and/or mutations arise, the invasive properties of SK-LU-1 cells can increase (38, 39). Most types of NSCLC express the protein marker nestin. In an attempt to quantitate total SK-LU-1 cell metastasis to the mouse lung, we utilized nestin immunoreactivity, as we and others have previously described (31, 40–43). We quantitated lung metastasis using an anti-human nestin antibody that does not react with mouse (31). Figure 5B indicates robust nestin immunoreactivity in the lung homogenates of HABP2 overexpressing but not vector control or HABP2 silenced mice. Quantitation of nestin immunoreactivity revealed ~10-fold increase in lung metastasis from HABP2 overexpressing primary tumors indicating the importance of this molecule in lung cancer progression (Figure 5C).

## Discussion

**Figure 6:**
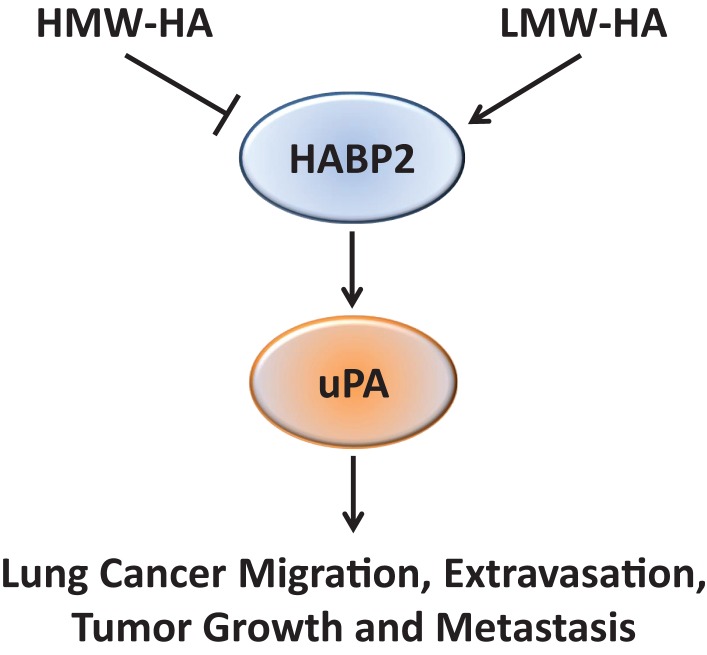
**A proposed schematic diagram illustrating the role of HA and HABP2 in lung cancer progression**. High-molecular weight hyaluronan (HMW-HA) inhibits, while low-molecular weight hyaluronan (LMW-HA) promotes HABP2 (extracellular serine protease upregulated in lung cancer) activity. Activated HABP2 can stimulate activation of urokinase plasminogen activator (uPA), which is required for LMW-HA-mediated lung cancer migration, transendothelial extravasation, tumor growth, and metastasis.

Based on the recent published data indicating HABP2 are increased in lung cancer (3), this study investigated the functional effects of HABP2 in human NSCLC cells both *in vitro* and *in vivo*. We observed that HABP2 overexpression in SK-LU-1 human NSCLC cells increased cell motion, migration, transendothelial extravasation, tumor growth and metastasis, and activation of the extracellular serine protease, uPA, implicated in cancer progression. In addition, LMW-HA enhances these *in vitro* activities and increases control and HABP2 overexpressing primary tumor volumes in nude mice. In contrast, HMW-HA either inhibited or had no effect on these processes. Importantly, the uPA inhibitor UK122 attenuated LMW-HA- and HABP2-dependent tumor cell motion, migration, and transendothelial extravasation. Taken as a whole, our data suggest that LMW-HA and HABP2 promote lung cancer progression through uPA-regulated pathways (Figure 6).

We have previously reported that HABP2, though primarily localized in the plasma, is upregulated in the lung endothelium with LPS-induced ALI and in cultured human pulmonary microvascular ECs (11). The enzymatic activity of HABP2 is differentially regulated by hyaluronan (HA) with HMW-HA (the main form of HA *in vivo*) inhibiting HABP2 protease activity, while LMW-HA (produced in disease states including cancer via HMW-HA cleavage by hyaluronidase enzymes and ROS) binds to the PABD of HABP2 and stimulates activity (11). Activated HABP2 induces protease-activated receptor signaling in EC, which leads to activation of the actin regulatory molecules RhoA and ROCK and endothelial barrier disruption. The barrier disruptive role of HABP2 were further confirmed by vascular silencing of HABP2 expression, which attenuated the vascular leakiness observed in LPS- and ventilator-induced lung injury (11). In this study, we have extended our findings to show that LMW-HA and HABP2 contribute to lung cancer progression in an uPA-regulated manner.

Hyaluronan binding protein 2 has been implicated in several disease processes, including atherosclerosis, ALI/ARDS, deep venous thrombosis, and cancer (3, 4, 6, 44). In addition, the G534E mutation of HABP2 is associated with cardiovascular disease and thromboembolism (45–47). However, the mechanism(s) by which HABP2 contributes to disease processes remains elusive. In normal blood coagulation cascade regulation, the role of HABP2 is complex since it activates factors involved in both coagulation and fibrinolysis (5). Our data that HABP2 promote uPA activation provides important mechanistic insights into HABP2 involvement in diseases associated with vascular dysfunction including lung cancer.

Identification of uPA as a target of HABP2 can have important clinical consequences. uPA is a extracelluar serine protease consisting of three domains, a serine protease domain, a kringle domain, and a growth factor-like domain (48). uPA is synthesized as a zymogen form and is activated by proteolytic cleavage by other proteases including HABP2 (7). Activated uPA has several targets including plasminogen and uPAR (49, 50). Recombinant uPA is used clinically as a thrombolytic agent in the treatment of severe deep venous thrombosis, pulmonary embolism, and myocardial infarction (51–53). However, elevated levels of uPA and other components of the plasminogen activation system are correlated with tumor malignancy (54). Through its interaction with uPAR, uPA has been shown to promote cancer cell adhesion, migration, and proliferation (55–59). These functions make uPA a potential drug target in cancer. Currently, small molecule serine protease inhibitors are being tested as a potential therapeutic strategy in various cancer types (60).

We have also discovered a differential effect of HA on HABP2 and uPA-regulated lung cancer progression based on its molecular weight. High levels of HA are observed in several types of lung cancer and inhibition of HA synthesis reduces lung cancer metastasis (12–17, 61, 62). Further, accumulation of LMW-HA is correlated with cancer invasion and metastasis (63). In addition, the enzymatic proteins to produce HMW-HA and LMW-HA are upregulated in lung cancer including hyaluronan synthase 3 and hyaluronidase-1 (64). Although other HA-binding proteins are upregulated in lung cancer including CD44, RHAMM, and LYVE-1 (65–69); to the best of our knowledge, this is the first report of a functional role of HABP2 in HA-mediated lung cancer progression.

Given previous published data indicating HABP2 is increased in lung cancer (3), we undertook a series of *in vitro* and *in vivo* experiments to examine the direct effects of HABP2 in lung cancer progression using human NSCLC cells. Our results indicate that LMW-HA and HABP2 promote lung cancer oncogenic properties. Importantly, our observations that HABP2 overexpression activates uPA suggest a potential therapeutic advantage using combinational HABP2 and uPA inhibitors in the treatment of NSCLC. Taken together, our data suggest a possible direct effect of HABP2 overexpression on lung cancer progression, and provide a potential functional explanation for the increased HABP2 expression observed in lung cancer. Our observations further suggest a possible therapeutic role for extracellular serine protease inhibitors, which merits further evaluation.

## Conflict of Interest Statement

The authors declare that the research was conducted in the absence of any commercial or financial relationships that could be construed as a potential conflict of interest.

## References

[1] Spiro SG Tanner NT Silvestri GA Janes SM Lim E Vansteenkiste JF Lung cancer: progress in diagnosis, staging and therapy. Respirology (2010) 15:44–50. 10.1111/j.1440-1843.2009.01674.x 20199634

[2] Stinchcombe TE Bogart J Veeramachaneni NK Kratzke R Govindan R Annual review of advances in non-small cell lung cancer research: a report for the year 2010. J Thorac Oncol (2011) 6:1443–50. 10.1097/JTO.0b013e3182246413 21709589

[3] Wang KK Liu N Radulovich N Wigle DA Johnston MR Shepherd FA Novel candidate tumor marker genes for lung adenocarcinoma. Oncogene (2002) 21:7598–604. 10.1038/sj.onc.1205953 12386823

[4] Kanse SM Parahuleva M Muhl L Kemkes-Matthes B Sedding D Preissner KT. Factor VII-activating protease (FSAP): vascular functions and role in atherosclerosis. Thromb Haemost (2008) 99:286–9. 10.1160/TH07-10-0640 18278176

[5] Romisch J. Factor VII activating protease (FSAP): a novel protease in hemostasis. Biol Chem (2002) 383:1119–24. 10.1515/BC.2002.121 12437095

[6] Sidelmann JJ Vitzthum F Funding E Munster AM Gram J Jespersen J. Factor VII-activating protease in patients with acute deep venous thrombosis. Thromb Res (2008) 122:848–53. 10.1016/j.thromres.2008.02.002 18394684

[7] Kannemeier C Feussner A Stohr HA Weisse J Preissner KT Romisch J. Factor VII and single-chain plasminogen activator-activating protease: activation and autoactivation of the proenzyme. Eur J Biochem (2001) 268:3789–96. 10.1046/j.1432-1327.2001.02285.x 11432747

[8] Choi-Miura NH Tobe T Sumiya J Nakano Y Sano Y Mazda T Purification and characterization of a novel hyaluronan-binding protein (PHBP) from human plasma: it has three EGF, a kringle and a serine protease domain, similar to hepatocyte growth factor activator. J Biochem (1996) 119:1157–65. 10.1093/oxfordjournals.jbchem.a021362 8827452

[9] Nakazawa F Kannemeier C Shibamiya A Song Y Tzima E Schubert U Extracellular RNA is a natural cofactor for the (auto-)activation of Factor VII-activating protease (FSAP). Biochem J (2005) 385:831–8. 10.1042/BJ20041021 15654766 PMC1134760

[10] Altincicek B Shibamiya A Trusheim H Tzima E Niepmann M Linder D A positively charged cluster in the epidermal growth factor-like domain of Factor VII-activating protease (FSAP) is essential for polyanion binding. Biochem J (2006) 394:687–92. 10.1042/BJ20051563 16332249 PMC1383718

[11] Mambetsariev N Mirzapoiazova T Mambetsariev B Sammani S Lennon FE Garcia JG Hyaluronic acid binding protein 2 is a novel regulator of vascular integrity. Arterioscler Thromb Vasc Biol (2010) 30:483–90. 10.1161/ATVBAHA.109.200451 20042707 PMC2825278

[12] Singleton PA. Hyaluronan regulation of endothelial barrier function in cancer. Adv Cancer Res (2014) 123:191–209. 10.1016/B978-0-12-800092-2.00007-1 25081530 PMC4470488

[13] Sironen RK Tammi M Tammi R Auvinen PK Anttila M Kosma VM Hyaluronan in human malignancies. Exp Cell Res (2011) 317:383–91. 10.1016/j.yexcr.2010.11.017 21134368

[14] Stern R Hyaluronan in cancer biology. Semin Cancer Biol (2008) 18:237 10.1016/j.semcancer.2008.03.017 18487061

[15] Tammi RH Kultti A Kosma VM Pirinen R Auvinen P Tammi MI. Hyaluronan in human tumors: pathobiological and prognostic messages from cell-associated and stromal hyaluronan. Semin Cancer Biol (2008) 18:288–95. 10.1016/j.semcancer.2008.03.005 18468453

[16] Toole BP Wight TN Tammi MI Hyaluronan-cell interactions in cancer and vascular disease. J Biol Chem (2002) 277:4593–6. 10.1074/jbc.R100039200 11717318

[17] Toole BP Hyaluronan: from extracellular glue to pericellular cue. Nat Rev Cancer (2004) 4:528–39. 10.1038/nrc1391 15229478

[18] Olczyk P Komosinska-Vassev K Winsz-Szczotka K Kuznik-Trocha K Olczyk K. [Hyaluronan: structure, metabolism, functions, and role in wound healing]. Postepy Hig Med Dosw (Online) (2008) 62:651–9. 19057507

[19] Wang A de la Motte C Lauer M Hascall V. Hyaluronan matrices in pathobiological processes. FEBS J (2011) 278(9):1412–8. 10.1111/j.1742-4658.2011.08069.x 21362136 PMC4401461

[20] Scott JE Heatley F. Biological properties of hyaluronan in aqueous solution are controlled and sequestered by reversible tertiary structures, defined by NMR spectroscopy. Biomacromolecules (2002) 3:547–53. 10.1021/bm010170j 12005527

[21] Girish KS Kemparaju K. The magic glue hyaluronan and its eraser hyaluronidase: a biological overview. Life Sci (2007) 80:1921–43. 10.1016/j.lfs.2007.02.037 17408700

[22] Furlan S La Penna G Perico A Cesaro A. Hyaluronan chain conformation and dynamics. Carbohydr Res (2005) 340:959–70. 10.1016/j.carres.2005.01.030 15780260

[23] Singleton PA Dudek SM Ma SF Garcia JG. Transactivation of sphingosine 1-phosphate receptors is essential for vascular barrier regulation. Novel role for hyaluronan and CD44 receptor family. J Biol Chem (2006) 281:34381–93. 10.1074/jbc.M603680200 16963454

[24] Lennon FE Mirzapoiazova T Mambetsariev N Mambetsariev B Salgia R Singleton PA. Transactivation of the receptor-tyrosine kinase ephrin receptor A2 is required for the low molecular weight hyaluronan-mediated angiogenesis that is implicated in tumor progression. J Biol Chem (2014) 289:24043–58. 10.1074/jbc.M114.554766 25023279 PMC4148838

[25] Lennon FE Singleton PA. Role of hyaluronan and hyaluronan-binding proteins in lung pathobiology. Am J Physiol Lung Cell Mol Physiol (2011) 301:L137–47. 10.1152/ajplung.00071.2010 21571904 PMC3154626

[26] Stern R. Devising a pathway for hyaluronan catabolism: are we there yet? Glycobiology (2003) 13:105R–15R. 10.1093/glycob/cwg112 14514708

[27] Stern R Kogan G Jedrzejas MJ Soltes L. The many ways to cleave hyaluronan. Biotechnol Adv (2007) 25:537–57. 10.1016/j.biotechadv.2007.07.001 17716848

[28] Stern R Asari AA Sugahara KN. Hyaluronan fragments: an information-rich system. Eur J Cell Biol (2006) 85:699–715. 10.1016/j.ejcb.2006.05.009 16822580

[29] Stern R Hyaluronidases in cancer biology. Semin Cancer Biol (2008) 18:275–80. 10.1016/j.semcancer.2008.03.017 18485730

[30] Lennon FE Mirzapoiazova T Mambetsariev B Poroyko VA Salgia R Moss J The Mu opioid receptor promotes opioid and growth factor-induced proliferation, migration and epithelial mesenchymal transition (EMT) in human lung cancer. PLoS One (2014) 9:e91577. 10.1371/journal.pone.0091577 24662916 PMC3963855

[31] Lennon FE Mirzapoiazova T Mambetsariev B Salgia R Moss J Singleton PA. Overexpression of the mu-opioid receptor in human non-small cell lung cancer promotes Akt and mTOR activation, tumor growth, and metastasis. Anesthesiology (2012) 116:857–67. 10.1097/ALN.0b013e31824babe2 22343475

[32] Mathew B Lennon FE Siegler J Mirzapoiazova T Mambetsariev N Sammani S The novel role of the mu opioid receptor in lung cancer progression: a laboratory investigation. Anesth Analg (2011) 112:558–67. 10.1213/ANE.0b013e31820568af 21156980 PMC4327979

[33] Hoffman RM Yang M Whole-body imaging with fluorescent proteins. Nat Protoc (2006) 1:1429–38. 10.1038/nprot.2006.223 17406431

[34] Maione P Rossi A Sacco PC Bareschino MA Schettino C Gridelli C. Advances in chemotherapy in advanced non-small-cell lung cancer. Expert Opin Pharmacother (2010) 11:2997–3007. 10.1517/14656566.2010.511615 20701554

[35] Beland MD Wasser EJ Mayo-Smith WW Dupuy DE. Primary non-small cell lung cancer: review of frequency, location, and time of recurrence after radiofrequency ablation. Radiology (2010) 254:301–7. 10.1148/radiol.2541090174 20032160

[36] Brachner A Sasgary S Pirker C Rodgarkia C Mikula M Mikulits W Telomerase- and alternative telomere lengthening-independent telomere stabilization in a metastasis-derived human non-small cell lung cancer cell line: effect of ectopic hTERT. Cancer Res (2006) 66:3584–92. 10.1158/0008-5472.CAN-05-2839 16585183

[37] Zhu M Gokhale VM Szabo L Munoz RM Baek H Bashyam S Identification of a novel inhibitor of urokinase-type plasminogen activator. Mol Cancer Ther (2007) 6:1348–56. 10.1158/1535-7163.MCT-06-0520 17431113

[38] Levy BP Drilon A Makarian L Patel AA Grossbard ML. Systemic approaches for multifocal bronchioloalveolar carcinoma: is there an appropriate target? Oncology (2010) 24(888–898):900. 21138169

[39] Toonkel RL Borczuk AC Powell CA Tgf-beta signaling pathway in lung adenocarcinoma invasion. J Thorac Oncol (2010) 5:153–7. 10.1097/JTO.0b013e3181c8cc0c 20101143 PMC2992959

[40] Chen Z Wang T Luo H Lai Y Yang X Li F Expression of nestin in lymph node metastasis and lymphangiogenesis in non-small cell lung cancer patients. Hum Pathol (2010) 41:737–44. 10.1016/j.humpath.2009.10.018 20132963

[41] Janikova M Skarda J Dziechciarkova M Radova L Chmelova J Krejci V Identification of CD133+/nestin+ putative cancer stem cells in non-small cell lung cancer. Biomed Pap Med Fac Univ Palacky Olomouc Czech Repub (2010) 154:321–6. 10.5507/bp.2010.048 21293543

[42] Krupkova O Jr Loja T Zambo I Veselska R. Nestin expression in human tumors and tumor cell lines. Neoplasma (2010) 57:291–8. 10.4149/neo_2010_04_291 20429619

[43] Ryuge S Sato Y Wang GQ Matsumoto T Jiang SX Katono K Prognostic significance of nestin expression in resected non-small cell lung cancer. Chest (2011) 139:862–9. 10.1378/chest.10-1121 20829334

[44] Wygrecka M Markart P Fink L Guenther A Preissner KT. Raised protein levels and altered cellular expression of factor VII activating protease (FSAP) in the lungs of patients with acute respiratory distress syndrome (ARDS). Thorax (2007) 62:880–8. 10.1136/thx.2006.069658 17483138 PMC2094251

[45] Ireland H Miller GJ Webb KE Cooper JA Humphries SE. The factor VII activating protease G511E (Marburg) variant and cardiovascular risk. Thromb Haemost (2004) 92:986–92. 15543324 10.1160/TH04-05-0275

[46] Hoppe B Tolou F Dorner T Kiesewetter H Salama A. Gene polymorphisms implicated in influencing susceptibility to venous and arterial thromboembolism: frequency distribution in a healthy German population. Thromb Haemost (2006) 96:465–70. 17003923

[47] Sedding D Daniel JM Muhl L Hersemeyer K Brunsch H Kemkes-Matthes B The G534E polymorphism of the gene encoding the factor VII-activating protease is associated with cardiovascular risk due to increased neointima formation. J Exp Med (2006) 203:2801–7. 10.1084/jem.20052546 17145954 PMC2118185

[48] Berdel W Wilhelm O Schmitt M Maurer J Reufi B Vonmarschall Z Urokinase-type plasminogen-activator (upa), a protease with cytokine-like activity in human hl-60 leukemic-cell line. Int J Oncol (1993) 3:607–13. 21573407 10.3892/ijo.3.4.607

[49] Archinti M Britto M Eden G Furlan F Murphy R Degryse B. The urokinase receptor in the central nervous system. CNS Neurol Disord Drug Targets (2011) 10:271–94. 10.2174/187152711794480393 20874700

[50] Kjaergaard M Hansen LV Jacobsen B Gardsvoll H Ploug M. Structure and ligand interactions of the urokinase receptor (uPAR). Front Biosci (2008) 13:5441–61. 10.2741/3092 18508598

[51] Collen D Fibrin-selective thrombolytic therapy for acute myocardial infarction. Circulation (1996) 93:857–65. 10.1161/01.CIR.93.5.857 8598075

[52] Forster A Wells P. Tissue plasminogen activator for the treatment of deep venous thrombosis of the lower extremity: a systematic review. Chest (2001) 119:572–9. 10.1378/chest.119.2.572 11171740

[53] Gurman P Miranda OR Nathan A Washington C Rosen Y Elman NM Recombinant tissue plasminogen activators (rtPA): a review. Clin Pharmacol Ther (2015) 97:274–85. 10.1002/cpt.33 25670034

[54] Mekkawy AH Morris DL Pourgholami MH. Urokinase plasminogen activator system as a potential target for cancer therapy. Future Oncol (2009) 5:1487–99. 10.2217/fon.09.108 19903074

[55] Allgayer H Heiss MM Riesenberg R Grutzner KU Tarabichi A Babic R Urokinase plasminogen activator receptor (uPA-R): one potential characteristic of metastatic phenotypes in minimal residual tumor disease. Cancer Res (1997) 57:1394–9. 9102229

[56] Bernstein AM Greenberg RS Taliana L Masur SK. Urokinase anchors uPAR to the actin cytoskeleton. Invest Ophthalmol Vis Sci (2004) 45:2967–77. 10.1167/iovs.04-0030 15326109

[57] Boonstra MC Verspaget HW Ganesh S Kubben FJ Vahrmeijer AL van de Velde CJ Clinical applications of the urokinase receptor (uPAR) for cancer patients. Curr Pharm Des (2011) 17(19):1890–910. 10.2174/138161211796718233 21711239

[58] Del Rosso M Margheri F Serrati S Chilla A Laurenzana A Fibbi G. The urokinase receptor system, a key regulator at the intersection between inflammation, immunity, and coagulation. Curr Pharm Des (2011) 17(19):1924–43. 10.2174/138161211796718189 21711238

[59] Deppe H Hohlweg-Majert B Holzle F Kesting MR Wagenpfeil S Wolff KD Content of urokinase-type plasminogen activator (uPA) and its inhibitor PAI-1 in oral mucosa and inflamed periodontal tissue. Quintessence Int (2010) 41:165–71. 20165748

[60] Froriep D Clement B Bittner F Mendel RR Reichmann D Schmalix W Activation of the anti-cancer agent upamostat by the mARC enzyme system. Xenobiotica (2013) 43:780–4. 10.3109/00498254.2013.767481 23379481

[61] Futamura N Urakawa H Arai E Kozawa E Ishiguro N Nishida Y. Hyaluronan synthesis inhibitor supplements the inhibitory effects of zoledronic acid on bone metastasis of lung cancer. Clin Exp Metastasis (2013) 30:595–606. 10.1007/s10585-012-9563-4 23288481

[62] Jiang P Li X Thompson CB Huang Z Araiza F Osgood R Effective targeting of the tumor microenvironment for cancer therapy. Anticancer Res (2012) 32:1203–12. 22493350

[63] Schmaus A Klusmeier S Rothley M Dimmler A Sipos B Faller G Accumulation of small hyaluronan oligosaccharides in tumour interstitial fluid correlates with lymphatic invasion and lymph node metastasis. Br J Cancer (2014) 111:559–67. 10.1038/bjc.2014.332 24937668 PMC4119989

[64] de Sa VK Carvalho L Gomes A Alarcao A Silva MR Couceiro P Role of the extracellular matrix in variations of invasive pathways in lung cancers. Braz J Med Biol Res (2013) 46:21–31. 10.1590/1414-431X20122263 23314337 PMC3854345

[65] Hwang J Kang MH Yoo YA Quan YH Kim HK Oh SC The effects of sonic hedgehog signaling pathway components on non-small-cell lung cancer progression and clinical outcome. World J Surg Oncol (2014) 12:268. 10.1186/1477-7819-12-268 25141859 PMC4155123

[66] Nunomiya K Shibata Y Abe S Inoue S Igarashi A Yamauchi K Relationship between serum level of lymphatic vessel endothelial hyaluronan receptor-1 and prognosis in patients with lung cancer. J Cancer (2014) 5:242–7. 10.7150/jca.8486 24665348 PMC3963081

[67] Augustin F Fiegl M Schmid T Pomme G Sterlacci W Tzankov A. Receptor for hyaluronic acid-mediated motility (RHAMM, CD168) expression is prognostically important in both nodal negative and nodal positive large cell lung cancer. J Clin Pathol (2015) 68:368–73. 10.1136/jclinpath-2014-202819 25731190

[68] Luo Z Wu RR Lv L Li P Zhang LY Hao QL Prognostic value of CD44 expression in non-small cell lung cancer: a systematic review. Int J Clin Exp Pathol (2014) 7:3632–46. 25120740 PMC4128975

[69] Man Y Cao J Jin S Xu G Pan B Shang L Newly identified biomarkers for detecting circulating tumor cells in lung adenocarcinoma. Tohoku J Exp Med (2014) 234:29–40. 10.1620/tjem.234.29 25175030

